# Atypical clinical presentation and long-term survival in a patient with optic nerve medulloepithelioma: a case report

**DOI:** 10.1186/1752-1947-6-123

**Published:** 2012-05-09

**Authors:** Natalia Pastora-Salvador, José Abelairas-Gómez, Jesús Peralta-Calvo, Eugenia García-Fernández, Carmen Morales-Bastos, Mónica Asencio-Durán, Fernando Carceller-Benito

**Affiliations:** 1 University Hospital La Paz, Paseo de la Castellana 261, 28046 Madrid, Spain

## Abstract

**Introduction:**

Medulloepithelioma is a rare congenital tumor of the primitive medullary neuroepithelium. A significant proportion of patients with medulloepithelioma arising from the optic nerve die from intracranial spread or cerebral metastasis. Because it has no known distinct clinical features and because of its low frequency, this tumor presents within the first two to six years of life and is usually misdiagnosed clinically as a different type of optic nerve tumor. Here, we describe a new and atypical case of medulloepithelioma of the optic nerve in a 12-year-old boy. To the best of our knowledge, he is the oldest reported patient to present with this disease and, now as an adult, has the longest documented period of disease-free survival.

**Case presentation:**

A 12-year-old Caucasian boy with headache and unilateral amaurosis was referred for a presumed optic nerve glioma to our hospital. A computed tomography scan showed optic nerve enlargement, and fundoscopy showed a whitish mass at the optic disc. Our patient had been followed at his local hospital for four years for an 'optic disc cyst' with no change or progression. He experienced mild progressive visual impairment during that period. He was admitted for resection, and a histopathological analysis revealed a medulloepithelioma of the optic nerve. Supplemental orbital radiotherapy was performed. He remained disease-free for 25 years.

**Conclusions:**

Medulloepithelioma of the optic nerve can clinically mimic more common pediatric tumors, such as optic glioma, meningioma, or retinoblastoma. Thus, medulloepithelioma should be included in the differential diagnoses of pediatric optic nerve lesions. Fundoscopy in these patients may provide relevant information for diagnosis. Anterior optic nerve medulloepitheliomas may behave differently from and have a better prognosis than medulloepitheliomas that have a more posterior location. Our case report illustrates that long-term survival can be achieved in patients with this malignant tumor.

## Introduction

Medulloepithelioma is a rare congenital tumor derived from the primitive medullary epithelium lining the neural tube. This is the most frequent congenital tumor of the ciliary body but is extremely infrequent in the optic nerve (ON) [1]. To the best of our knowledge, only 10 cases of medulloepithelioma of the ON have been reported to date [2-12]. This rare tumor usually presents within the first two to six years of life and is considered to be clinically malignant and entails significant vital compromise and mortality because of intracranial spreading or cerebral metastasis [2]. The characteristic clinical features and prognosis of these rare tumors are unknown. Here, we describe a new and atypical case of medulloepithelioma of the ON in a 12-year-old boy. To the best of our knowledge, he is the oldest reported patient to present with this disease and, now as an adult, has the longest documented period of disease-free survival.

## Case presentation

A 12-year-old Caucasian boy was referred with a presumed diagnosis of right ON glioma to our hospital. Four years earlier, his local ophthalmologist had diagnosed a 'yellow-whitish, well-demarcated papillary cyst with central retinal vessel displacement' in his right eye (oculus dexter, or OD) and documented a visual acuity of 20/30 in both eyes. He was examined twice a year, and his vision remained stable. Four years later, his ophthalmologist documented 'visual acuity of 20/80 and concentric visual field reduction to the central 10° isopter in OD'. Fundoscopic variation of the lesion was not noted.

One month later, after a severe headache, visual loss progressed and no light perception with an absolute afferent pupillary defect was present in the OD. A computed tomography (CT) scan showed right ON thickening, and the boy was referred and admitted to our center.

**Figure 1:**
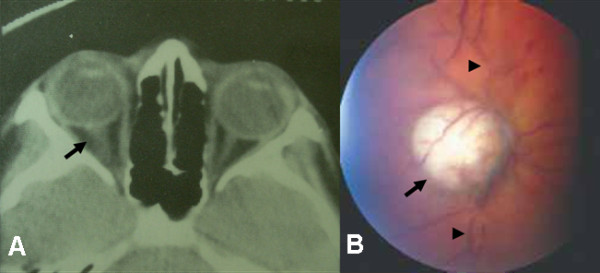
**Clinical presentation and computed tomography images of a 12-year-old Caucasian boy**. (A) His right optic nerve appears elevated and enhanced on computed axial tomography (arrow). His left optic nerve appears normal. (B) A well-defined whitish mass at the optic disc (arrow) with tortuous and dilated retinal vessels (arrowhead) by fundoscopy.

At admission, the described neuro-ophthalmologic findings were confirmed, and on CT, the optic disc appeared elevated and enhanced after intravenous contrast was administered. Fundoscopy revealed a well-defined whitish mass without macroscopic calcification at the ON head, dilated and tortuous retinal vessels, and small superficial retinal hemorrhages (Figure 1). A complete general examination did not detect any other alterations.

After informed consent was obtained from our patient's parents, a right transfrontal exploratory orbitotomy with canalicular unroofing was performed, and a normal chiasm, prechiasmatic and canalicular ON, and grayish infiltration in the proximal intraorbital portion were revealed. A frozen intraoperative biopsy of the tumor was diagnosed as an 'unrecognized tumoral process', and 0.8 cm of distal ON was resected. Because the eye had no light perception and the tumor was potentially malignant, the remaining ON and globe were enucleated with excision of the retrobulbar orbital fat.

**Figure 2:**
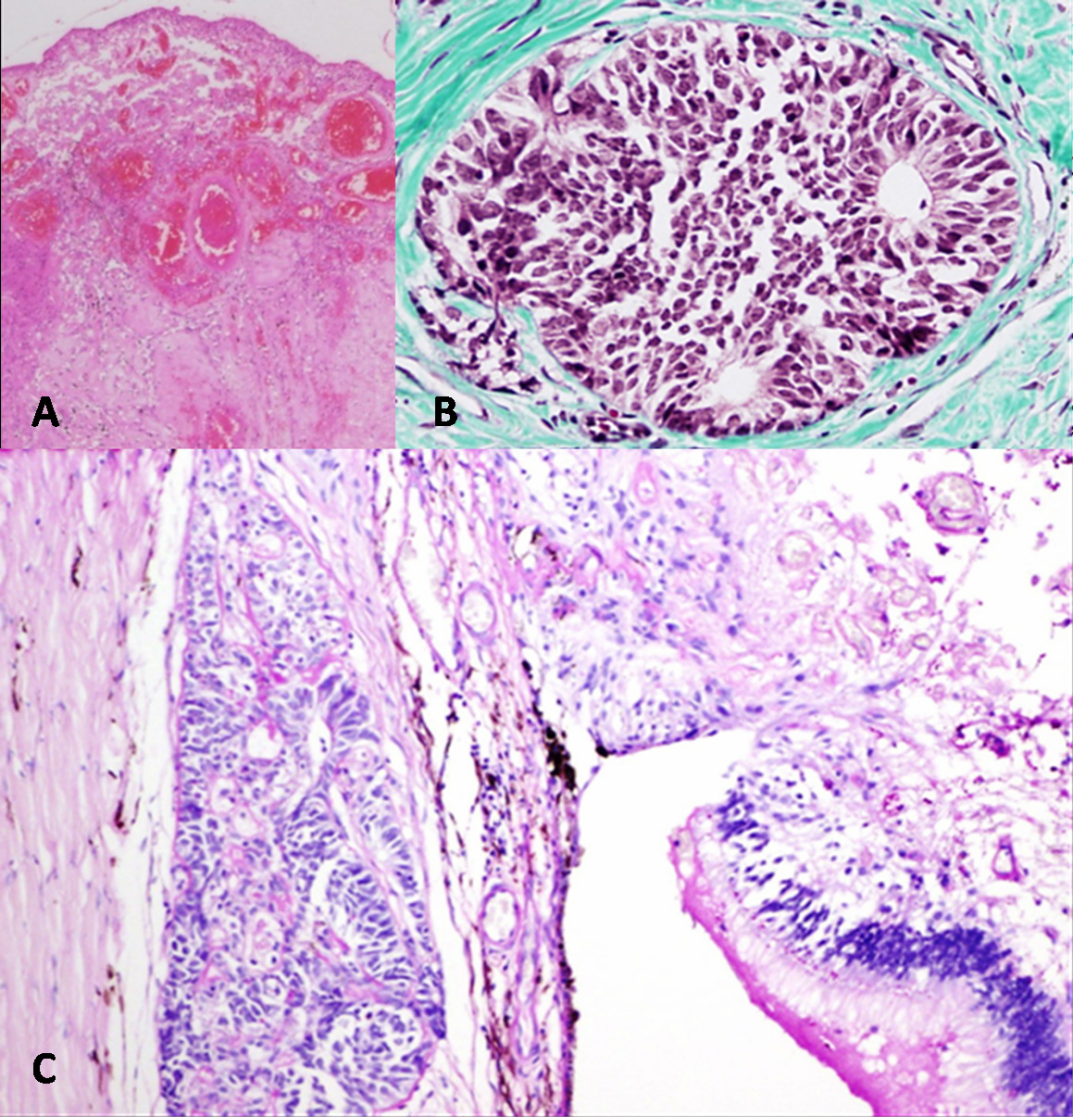
**Histopathology of malignant non-teratoid medulloepithelioma of the optic nerve**. (A) The lesion at the optic disc has a central necrotic area with numerous dilated vessels of thin endothelium surrounded by Fontana-Masson-positive spindle cells with pigment. (B) A neoformation of poorly differentiated neuroblastic cells with scanty cytoplasm extends to both sides under the retina at the root of the optic nerve. Tumoral cells form small solid nests and rosette-like structures, have wider cytoplasm with a vesicular nucleus, and show cystic-degenerative changes with an eosinophilic, PAS (periodic acid-Schiff)-positive amorphous material in the center. (C) Nests of tumoral cells are distinguished under the retina and infiltrate the sclera. Stains: hematoxylin-eosin (A, C) and Masson trichrome (B). Magnifications: 40 × (A), 400 × (B), and 100 × (C).

A gross examination of the eye revealed an ON with a width of 6 to 7 mm and a length of 6 mm. A sagittal section through the globe revealed a small, whitish, rounded tumor of 2 to 3 mm with a soft surface at the optic disc. No lesions of the cornea, iris, lens, or ciliary processes were detected. The tumor had areas composed of poorly differentiated neuroblastic cells with nuclear pleomorphism but no mesenchymal elements. Thus, the final histopathological diagnosis was malignant non-teratoid medulloepithelioma of the ON [13]. There was dense granulation tissue with giant cells, but no tumor was present in the adipose tissue surrounding the ON (Figure 2).

Systemic work-up was negative for any evidence of metastatic disease. In view of the significant mortality rate of this tumor, orbital exenteration was suggested, but our patient's parents declined [3-5]. Fifty gray of telegammatherapy (Co^60^) was applied in two fields. Our patient was closely followed with annual orbital CT and half-yearly echography for six years without evidence of recurrence. At present, he is 37 years old and has a disease-free survival of 25 years.

## Discussion

Medulloepithelioma of the ON remains a challenge because of its low frequency. The clinical presentation is similar to that of other neoformations of the ON during childhood. When its location involves the orbital portion of the ON, proptosis and optic disc swelling may occur; lesions that are more posterior can produce a progressive retrobulbar optic neuropathy [1]. Interestingly, medulloepitheliomas that originate from an anterior location can exhibit a mass at the ON head on fundoscopic examination, and this may be a distinct clinical feature that aids in the differential diagnosis because intraocular extension is very infrequent in other tumors arising from the ON [1-3,5-8].

Differential diagnoses of growing ON masses with reduced visual function and an intraocular component should include glioma, retinoblastoma, medulloepithelioma, and meningioma. Glioma and retinoblastoma with ON infiltration are the most frequently presumed diagnoses. Other processes, such as intraocular lesions extending onto the ON (melanocytoma, melanocytic tumors, astrocytoma, or retinal pigment epithelium proliferations), metastasis (infiltration from hematologic malignancies), or inflammatory disorders (mainly granulomatosis), are rare but should also be properly ruled out.

There are no distinct neuroimaging features of medulloepitheliomas of the ON. Calcification can be detected, as in retinoblastoma [14]. In addition, primary ON sheath meningiomas in children can present with severely reduced vision, intraocular and intracranial invasion, and calcification detected by neuroimaging methods [15].

In patients with medulloepitheliomas arising from the posterior ON, mortality is usually caused by untreatable intracranial invasion or cerebral metastasis [3,4,9,10]. In contrast, medulloepitheliomas arising from the anterior ON may have a better prognosis because a greater proportion of them are symptomatic, leading to earlier diagnosis [2,4-7,11], and they have a smaller tendency to extend into the cranium [8]. An anterior origin may be associated with the possibility for intraocular extension that, in turn, reduces the tendency of the tumor to extend into the cranium. Moreover, localized intraocular tumors may have no direct connection to cerebrospinal fluid that allows tumors to spread. Thus, in these cases, an anterior (intraocular) approach to lesion biopsy can be considered if there is a reasonable diagnostic and therapeutic dilemma [8].

Our patient was remitted with presumed glioma of the ON. However, the white mass in the optic disc was not typical for a glioma, and the four-year history of a growing papillary mass was not typical for a malignant process. This could be caused by low aggressiveness of the tumor or late malignant transformation [6]. Despite the unusualness of this presentation, this patient was successfully managed with surgery and adjuvant radiotherapy and maintained a disease-free survival of 25 years, which, to the best of our knowledge, is the longest reported to date.

The most effective management of medulloepitheliomas of the ON remains unclear. It seems that total excision is curative but, because of extension to adjacent structures, is not always possible. Adjuvant chemotherapy, radiotherapy, or extensive resection can be effective in cases of tumor infiltration at the resection margin [12], but there is no consensus about the regimen.

## Conclusions

ON alterations with continued, even mild, visual loss in children should be considered for neuroimaging. Medulloepithelioma of the ON should be included in the differential diagnoses of ON masses. In addition to ON enlargement, an intraocular component (papillary mass) may be present. Surgery is the first and most important therapeutic step, but the need for associated adjuvant treatments, even without residual disease, and the most effective treatment still cannot be specified. The long-term prognosis of these rare tumors is still unknown. However, our case report reveals, for the first time, that successful treatment can achieve a long-term survival.

## Abbreviations

CT: computed tomography; OD: oculus dexter (right eye); ON: optic nerve.

## Consent

Written informed consent was obtained from the patient for publication of this case report and accompanying images. A copy of the written consent is available for review by the Editor-in-Chief of this journal.

## Competing interests

The authors declare that they have no competing interests.

## Authors' contributions

NP-S, JP-C, and MA-D performed examinations, interpreted data regarding clinical and neuroimaging findings of the patient, and contributed to the conception and design of the manuscript. JA-G and FC-B operated on the patient and made major contributions to the writing of the manuscript. EG-F and CM-B performed the histological examination of the lesion and wrote the text regarding the histopathological findings. All authors read and approved the final manuscript.
